# Resolving the Haplotype Complexity of Colorectal Cancer Genomes with Droplet Barcode Sequencing

**DOI:** 10.3390/life16060874

**Published:** 2026-05-22

**Authors:** Humam Siga, Pontus Höjer, Parham Pourbozorgi, Hooman Aghelpasand, Max Käller, Johan Hartman, Cecilia Williams, Afshin Ahmadian

**Affiliations:** 1 KTH Royal Institute of Technology, Department of Gene Technology, SciLifeLab, 171 21 Solna, Sweden; humam.siga@scilifelab.se (H.S.); pontus.hojer@scilifelab.se (P.H.); hooman.aghelpasand@scilifelab.se (H.A.); max.kaller@cellanome.com (M.K.); 2 Karolinska Institute, Department of Oncology-Pathology, 171 77 Stockholm, Sweden; johan.hartman@ki.se; 3 KTH Royal Institute of Technology, Department of Protein Science, SciLifeLab, 171 21 Solna, Sweden; cecilia.williams@scilifelab.se

**Keywords:** cancer genomics, colorectal cancer, CRC, linked-reads, haplotyping, droplet barcode sequencing, DBS, structural variants, somatic mutations

## Abstract

Precision medicine is increasingly applied in the cancer clinic, adapting treatment to genomic alterations of the tumor. However, whether alterations disrupt the function of a protein can depend on if both alleles of a gene are altered. While massively parallel sequencing technologies can identify sequence aberrations, they are limited in resolving the corresponding haplotype information. In this proof-of-concept case study, we applied the linked-read droplet barcode sequencing (DBS) technology to resolve the haplotype complexity of colorectal cancer genomes on paired tumor and normal samples. Several cancer-related genes carried multiple mutations in either one or both haplotypes. Additionally, a number of haplotype-resolved large structural variants and copy number alterations were detected and phased with short somatic variants. Nearly all characterized oncogenic pathways harbored some of the identified short somatic variants. The study demonstrates that linked-read DBS technology can characterize complex genetic variations in a haplotype context and may provide essential information for personalized approaches.

## 1. Introduction

Cancer remains a leading cause of death worldwide, with colorectal cancer (CRC) ranked as the second most common cause of cancer mortality [1]. CRC is a complex and heterogeneous disease characterized by a multitude of genetic alterations [2]. Knowing whether these genetic alterations are affecting both alleles is critical for precision medicine, as the affected protein function can depend on this information. Despite significant advances in the understanding of CRC, many aspects of its genetic complexity remain elusive [3]. Whole genome sequencing (WGS) using massively parallel sequencing (MPS) platforms has revolutionized the study of complex cancer genomes. The complexity can manifest itself in small or large variations, spanning from single-nucleotide variants (SNVs) to large alterations in the structure of the genome, known as structural variants (SVs). SVs may cause copy number alterations (CNAs) such as deletions, duplications, or insertions. Alternatively, they can cause genomic aberrations that are copy-neutral, as in inversions or translocations [4].

Characterizing complex genetic variations in specific haplotypes to reveal the combined effect of alterations presents a substantial challenge. Short-read MPS technologies, commonly used for WGS studies, lack the capability of addressing questions related to the long-range haplotype information required to comprehensively characterize the cancer genome [5]. To address the issue of gaining long-range genomic information, linked-read and long-read sequencing technologies have emerged in the past decade as the two main approaches. However, using long-read sequencing for WGS of the human genome is considerably more expensive and less accessible compared to short-read sequencing methods [6]. Additionally, larger input amounts of DNA are generally required for library generation, and some long-read approaches still have issues related to read qualities [7]. Linked-read technologies, on the other hand, offer a promising solution to address these issues, providing a powerful tool to accurately resolve haplotypes and unravel the mutational landscape of the sample. Linked-read sequencing combines short-read sequencing with long-range information, which enables the identification and reconstruction of haplotypes. This approach leverages compartment-based barcoding to tag individual DNA molecules from the same genomic region, allowing for phasing complex genomic variations. By preserving long-range information, linked-read sequencing offers insights into the structural and functional organization of the genome, enabling the detection and phasing of novel variants, including SNVs, insertions, deletions, CNAs, and other SVs. While a few linked-read sequencing technologies are available [8,9,10,11,12,13], these are mostly provided by private vendors, and the adoption and wide use of these techniques in cancer studies have been greatly hampered by the cost of library preparation and by the quality of the data.

We have previously presented an alternative linked-read method targeted for genome-wide haplotyping analysis, named droplet barcode sequencing (DBS) [14,15]. Moreover, we have employed this method for DNA-assisted protein profiling on magnetic particles [16], and for surface markers of individual extracellular vesicles [17]. DBS libraries can be prepared cost-effectively without the need for advanced microfluidic devices. In addition, high-quality haplotypes can also be obtained from relatively degraded DNA samples. This is highly beneficial for cancer studies, where the integrity of the extracted genomic DNA is often compromised. Thus, the DBS technique holds an immense potential for accurately characterizing somatic mutations (SMs) in a haplotype context within cancer samples.

CRC exhibits substantial genetic heterogeneity, with a high rate of mutational events contributing to tumorigenesis and progression [18]. This makes it an ideal representative case to study. In this proof-of-concept work, we show that DBS technology provides an opportunity to unravel the complexity of CRC genomes. By capturing long-range genomic information, we demonstrate that this technology can accurately reconstruct the haplotypes, facilitating the identification of co-occurring mutations and their association with key structural variants, as well as highlighting key genes that are likely to be influenced by these aberrations.

## 2. Materials and Methods

### 2.1. Sample Collection

Two colorectal tumors and matched noncancerous adjacent tissue were collected from two female patients, aged 77 (#18) and 88 (#19), undergoing surgery in Stockholm after informed consent. Both tumors were low-grade adenocarcinoma (TNM stage pT2), one node-negative (N0, #18) and one node-positive (N1a, #19). The specimens were placed in RNAlater and frozen at −80 °C. Corresponding transcriptomes have been analyzed and published previously [19]. The study was approved by the regional ethical review board in Stockholm, Sweden.

### 2.2. DBS Library Preparation

HMW gDNA was recovered from these tissue samples using the MagAttract HMW DNA Kit (Qiagen, Hilden, Germany #67563) according to the manufacturer’s instructions. The fragment size was analyzed using the Femto Pulse instrument (Agilent Technologies, Santa Clara, CA, USA) and the DNA concentration was determined using a Qubit assay. DBS libraries were generated as previously described [6,14]. For patient P18, two libraries were generated from each normal and tumor sample, while for patient P19, three libraries were generated. Final libraries were sequenced using Illumina NovaSeq with an S4 2 × 150 bp kit (Illumina Inc., San Diego, CA, USA).

### 2.3. Phasing

Each DBS dataset was run through BLR (for details on running BLR, see ref. [6]) mapping to GRCh38 using EMA [20] (v.0.6.2). The processed BAMs were then merged for each sample and patient for downstream analysis. Using the merged BAMs, variants were called with DeepVariant [21] (v.1.3.0). Phasing was performed on heterozygous SNPs using HapCUT2 [22] (v.1.3.2).

### 2.4. Titan CNA Calling

Mapped CRAM files tagged with the phasing information, along with the called variants, were used from BLR output as inputs for the Titan pipeline (https://github.com/HSiga/TitanCNA_10X_snakemake/tree/working, commit #cbc80cc9, accessed on 17 May 2026) using the 10× workflow with adjustments. The config file was adjusted as follows: the optional snpVCF reference file was commented out, sex was set to ‘female’, bx_mapQual was set to 20, het_minVCFQuality to 30, and a reference genome path was set. The script getTumourAlleleCountsAtHETSites.py was adjusted for the proper input from the VCF files.

### 2.5. ASCAT CNA Calling

The analysis was run according to the workflow from the Maftools (v2.16.0). Files sample#.nucleotide_counts.tsv were first generated using the gtMarkers function, after the modification to read CRAM files, and then the prepAscat function was used to generate the main input files to run ASCAT. ASCAT (v3.1.2) was then applied to process the input files and plot the results.

### 2.6. CNVkit CNA Calling (RNA-seq)

Copy number analysis on RNA-seq data was performed using CNVkit [23] (v0.9.10) according to the tool recommendations. For the RNA-seq data, raw read counts were acquired from an earlier study [19]. These non-normalized transcript counts were used as input, along with pre-calculated CNV-expression correlation coefficients bundled with CNVkit. Using this, the command ‘cnvkit.py import-rna’ was used to generate normalized and bias-corrected data. This data was then segmented using ‘cnvkit.py segment’ with option ‘-m cbs’ to use the circular binary segmentation (CBS) method [24,25]. The values were then centered using ‘cnvkit.py call’ with options ‘-m none --center’.

### 2.7. Somatic SV Analysis

Structural variants (SVs) were called with a modified version of NAIBR [26] (https://github.com/pontushojer/NAIBR, v0.5.1, accessed on 17 May 2026) as a part of the BLR pipeline. In addition, structural variants were also called using LinkedSV [27] (v1.0.1) (https://github.com/HSiga/LinkedSV/, commit #84186a9, accessed on 17 May 2026). LinkedSV was run in whole-genome mode using the ‘--somatic_mode’ option for the tumor samples and the ‘--germline_mode’ option for the normal samples. From the LinkedSV calls, only the small deletions (file *.small_deletions.bedpe) and filtered large SVs (file *.filtered_large_svcalls.bedpe) were used.

All SVs labeled as “PASS”, i.e., passing filters, from NAIBR and LinkedSV were combined for each patient. This union set for each patient was then re-scored using NAIBR for both the corresponding tumor and normal sample. The re-scored SVs were then merged for each patient with BCFtools [28] (v1.17) using the subcommand “merge” with options “-m none -f PASS”. Similar SVs were collapsed using Truvari [29] (v v3.2.0) with the subcommand “collapse” and parameters “--chain -k maxqual --pctsim 0 --pctovl 0.8 --pctsize 0.8 --refdist 1000 -S 100_000”. These SVs were then filtered with SURVIVOR [30] (subcommand “filter”) for size (2–100 kb) and against a BED of blacklisted regions (https://github.com/10XGenomics/longranger/raw/master/tenkit/lib/python/tenkit/sv_data/10X_GRCh38_no_alt_decoy/default_sv_blacklist.bed, accessed on 17 May 2026) expanded by 10 kb with BEDtools [31] (subcommand “slop”). We further filtered out SVs whose breakpoints intersected a segmental duplication within 20 kbp (https://github.com/10XGenomics/longranger/raw/master/tenkit/lib/python/tenkit/sv_data/10X_GRCh38_no_alt_decoy/default_segdups.bedpe, accessed on 17 May 2026). From this filtered set of SVs, the SVs only called by the tumor sample were used as the somatic set. These SVs were then visualized with Samplot [32] (#f4490b2) for a final manual filtration.

### 2.8. Barcode Span Visualization

An in-house developed tool was used to plot the selected SVs, named haploplot (https://github.com/HSiga/haploplot, v0.1.0, accessed on 17 May 2026). For deletions, a span of 30 kbp before the start of the deletion start site and 30 kbp after the deletion end site was selected. Only shared unique barcodes in these two regions were kept (spanning barcode list). The plotted region was then divided into windows (5 kbp) and the number of intersected barcodes with the spanning barcode list was plotted per haplotype. Barcodes in unphased reads were ignored.

For inversions, 500 bp flanking each of the break points were selected. Unique barcodes intersecting both break points were intersected. The plotted region was then divided into windows (500 bp) and the number of barcodes shared by both the break points was then plotted. Barcodes in unphased reads were ignored.

### 2.9. Somatic Variant Analysis

Somatic variants were called using LANCET (https://github.com/nygenome/lancet, v1.1.0, accessed on 17 May 2026) for each of the paired tumor/normal samples, for each chromosome in parallel. Variants with the label “PASS” were then selected and annotated using vcf2maf [33] tools (https://github.com/mskcc/vcf2maf, v1.6.20, accessed on 17 May 2026) with Ensembl Variant Effect Predictor (VEP) [34] (v.102.0). Annotations with vcf2maf with VEP were done following the instructions from the Mutscape toolkit [35]. Maf files (mutations annotation format) were then processed in R (v.4.1.2) using Maftools [36] (https://github.com/PoisonAlien/maftools, v2.17.0, accessed on 17 May 2026) following the package instructions and documentation, with some adjustment of the codes accounting for the haplotype information reported by LANCET. COSMIC V98 [37] gene symbols were used for the intersection with the gene symbols from the annotated Maf mutations. Phred-scaled *p*-value of Fisher’s exact test for ref/alt haplotype counts in the tumor (HPST) ≥ 30 was used as a threshold to filter out low-quality phased SMs in the tumor. GenVisR (https://github.com/griffithlab/GenVisR, v1.26.0, accessed on 17 May 2026) was used to plot the local mutations, with adjustment to account for haplotype information, and the CopyNumberPlots R package (https://github.com/bernatgel/CopyNumberPlots, v1.10.0, accessed on 17 May 2026) was used and modified to plot the coverage plot with the mutated genes.

### 2.10. RNAseq Data Processing

Raw read counts were acquired from an earlier study [19]. Samples P10610_116, P10610_117, P10610_129, P10610_130 were selected for T18, T19, N18_Proximal, N19_Proximal respectively. Genes with low counts (<10 in the sum of all the samples) were filtered out. Reads were then normalized using DESeq2 (v1.34.0) [38], and reannotated with GRCh38 Ensembl gene IDs (gencode.v49.annotation.gtf).

### 2.11. RNAseq Data Integration with Mutations

The normalized gene counts list was used for the downstream analysis. To estimate the relative expression per sample, only genes with sufficient counts per sample were kept (N18 + T18 ≥ 5 or N19 + T19 ≥ 5). Log2 expressions were then calculated per patient (T18 + 0.01/N18 + 0.01 and T19 + 0.01/N19 + 0.01). Only genes in Chromosomes (1–22, X) were kept. RNA expression data was then intersected with mutated Cosmic genes that had 2 or more mutations (using gene names).

### 2.12. RNAseq Data Integration with Copy Number Analysis

Copy number results for the optimal solutions as reported by Titan, and mutations in genes that had 2 more mutations were intersected. Plots were generated using the R packages karyoploteR (v1.20.3) [39] and CopyNumberPlots (v1.10.0) [40], with modifications to include the RNA expression data of the mutated genes. The log2 fold expression was set to max 3 and min −3 if the gene expression exceeded these numbers, and when no log2 fold expression was available, it was set to 0.

### 2.13. Resource Verification

Some parts of the codes were developed with the assistance of AI tools; all contributions were reviewed and validated by the authors.

All referenced GitHub links were to be accessible and functional as of 20 May 2026.

## 3. Results and Discussion

**Figure 1:**
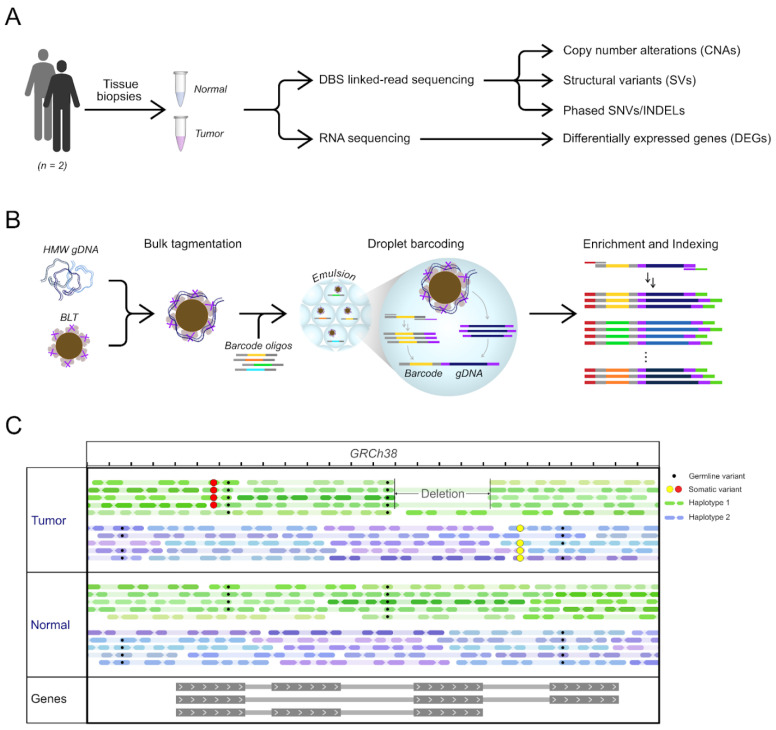
(**A**) Study design. DNA from paired tumor and normal samples undergoes linked-read library preparation through DBS technology to call unique genomic alterations in the tumor sample. The samples are also RNA sequenced to compare transcript profiles to CNA. (**B**) Overview of the DBS method for generating linked reads. (**C**) Example of analysis enabled by the workflow. Barcoded reads are mapped to GRCh38 as a reference, and haplotypes are generated by using germline variants. Unique somatic variants, SVs and CNA, are phased in the cancer genome.

Paired tumor (T) and proximal normal (N) tissue biopsies were acquired from two CRC patients, henceforth referred to as P18 and P19. Genomic DNA was extracted from these samples and underwent whole-genome linked-read sequencing using the DBS method. The use of paired tumor-normal samples allows for the detection of unique genetic alterations accumulated during tumorigenesis, and the linked reads maintain the phasing information to extrapolate the haplotypes. In addition to the genomic data, corresponding RNA-seq data were used, generated in an earlier study [19]. Figure 1A illustrates the workflow of the data and experiments utilized in this study, and Figure 1B gives an overview of the DBS method for generating linked-read libraries [14]. In brief, high molecular weight genomic DNA (HMW gDNA) is mixed with bead-linked transposases (BLT). HMW gDNA wraps around the BLT, forming an on-bead tagmentation complex (BLT-gDNA). The complexes are then distributed across millions of emulsion droplets together with barcoding oligonucleotides. In each droplet, adaptor sequences are introduced throughout the gDNA fragments, and a unique DNA barcode is amplified and then coupled to the adaptor-linked fragments. This process allows labeling of all fragments originating from the same HMW gDNA molecule with a unique droplet barcode (DBC). After emulsion breakage, the barcoded DNA molecules are enriched, followed by library indexing and high-throughput short-read sequencing. Subsequently, the resulting linked-read data are computationally processed and haplotypes for each sample are resolved, enabling phasing of somatic alterations (Figure 1C).

DBS linked-reads from both patients and tissues were processed using our Barcode Linked Reads (BLR) pipeline [6]. The pipeline maps the reads to the human reference genome, using EMA alignment software [20] and then calls small variants (SNPs and small insertions or deletions (small indels)) using DeepVariant [21]. Heterozygous variants are then phased using HapCUT2 [22]. These phased variants are used to assign mapped reads to each of the haplotypes. BLR reported a lower coverage in patient P18 at around 30× compared to 46× for P19 (Supplementary Table S1). The inferred molecule length was between a median of 8.4 and 11.4 kilobase pairs (kbp). DeepVariant called 2.119 to 2.210 million heterozygous SNVs, of which around 99.6% were phased. The phase block lengths had an NG50 from 1.3 to 2.7 megabase pairs (Mbp) (Supplementary Table S1). The BLR output was then used to investigate somatic alterations at multiple scales. Starting with CNA analysis, the TitanCNA linked-read workflow [41,42] was performed on both paired samples. Large structural variants were then called using NAIBR [26] and LinkedSV [27]. Finally, joint variant calling for small somatic variants (point mutations and small indels) was done using LANCET [43,44].

**Figure 2:**
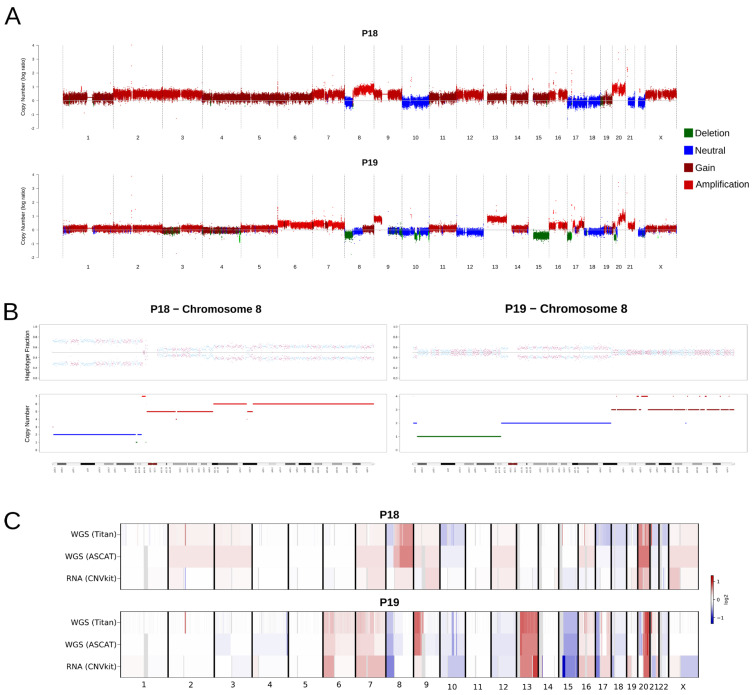
Copy number analysis for both patients. (**A**) Copy numbers called by Titan. (**B**) Haplotype fraction and copy number for chromosome 8. Haplotype fraction colors represent different phase blocks. (**C**) Correlation of logR ratios generated by Titan and ASCAT on WGS data and CNVkit of RNA-seq data for patients P18 and P19. Gray bands indicate non-segmented regions.

The CNA results revealed large chromosomal copy number aberrations in both tumor samples (Figure 2A). The average tumor ploidy was estimated at 2.96 for P18 and 2.16 for P19, with a purity of 44% and 54%, respectively. Both samples shared multiple copy number signatures, several of which have been previously reported for CRC [45,46]. For example, gains in chr20q are common in CRC and likely important for the tumor progression into carcinoma [47]. Another commonly reported CNA that was detected was the gain in chromosome 8q, harboring, for instance, the proto-oncogene MYC. The additional information of DBS data allows the linked reads to be phased into their originating alleles, as reads in the same phase block are likely to have originated from the same allele. The TitanCNA algorithm calculates the estimated copy number of a large span of segments on each of the chromosomes. Figure 2B shows phase block spans and the estimated copy numbers of chromosome 8 in both patients. In P18, most of the short arm (8p) shows a diploid copy number (*n* = 2) but the haplotype fraction exhibits a large imbalance. This suggests a copy-neutral loss of heterozygosity (cnLOH) on 8p, which is explained by a deletion in one allele and a duplication in the other. In contrast, the long arm (8q) in the same patient has an increased copy number with an imbalance in the copy number fraction. In P19, the results suggest a wide copy number loss (CN = 1) in 8p. The haplotype fraction is relatively centered on 0.5. The results suggest a homozygous deletion in 8p, knowing that the purity of the tumor is about 54%, the admixture of normal cells in the tumor sample compensates for the expected allelic imbalance.

To validate the CNA results from Titan, we performed additional CNA analysis using ASCAT [48] on the WGS data, and CNVkit [23] on the RNA-seq data (Figure 2C). Compared to ASCAT, the CNA pattern was highly similar. For the RNA-seq-based CNAs, there were several similarities between the expression profile compared to the WGS-based calls, as in chromosomes 7, 10 and 18 in both patients. The correlation between CNA and differential gene expression has previously been reported in a pan-cancer study [49].

**Figure 3:**
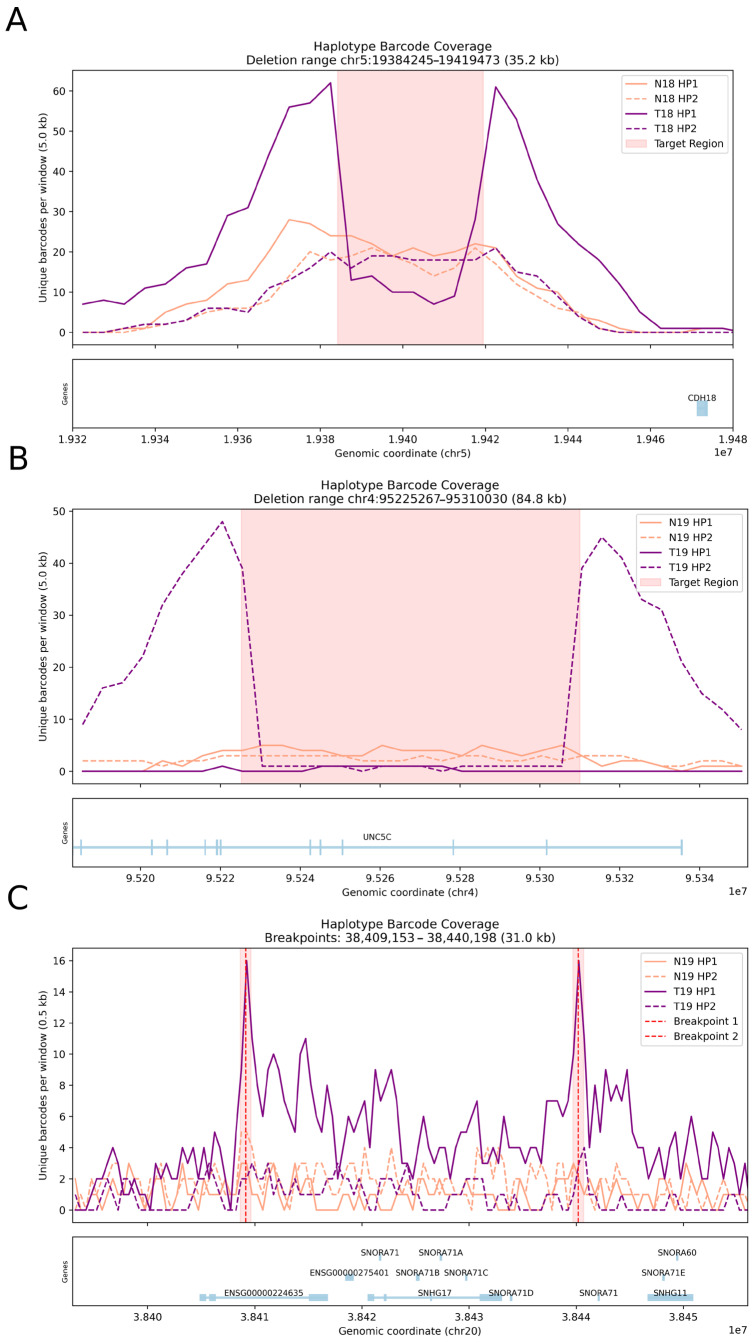
Examples of large somatic SVs. (**A**) 35 kbp deletion in P18 highlighted with light red, located on chr5 about 50 kbp downstream of the *CDH18* gene. (**B**) 85 kbp deletion in P19 on chr4 that overlaps *UNC5C* gene. (**C**) 81 kbp inversion in P19 located on chr20 that overlaps the *SNHG17* gene. Visualization was generated with haploplot.

Large structural variants were called using both NAIBR [26] and LinkedSV [27]. For the SVs unique to the tumor, visual inspection revealed that many were not called in the normal samples. To reduce false positives, we created a union set of SVs for each patient and re-scored it with NAIBR against the respective normal and tumor reads. This yielded around 1700 SVs per sample (Supplementary Table S2). These were then merged per patient and collapsed to remove overlapping calls and finally filtered for size (2–100 kbp) and against blacklisted regions. Choosing 2 kbp as a lower threshold was due to the low reliability of smaller SVs, as shown in our previous study [6]. Out of the filtered set, 24 and 42 SVs were called in tumor samples of P18 and P19, respectively. These SVs were then visually inspected using Samplot [32], removing any calls with unclear evidence or missed germline calls. This left 9 SVs for P18 and 16 SVs for P19. A selection of these SVs is shown in Figure 3, where we plot the spanning barcodes over the deletion regions or the inversion break points (see Section 2.8: Barcode span visualization). For P18, a 35 kbp deletion was found on chr5, 50 kbp downstream from the *CDH18* gene (Figure 3A). Interestingly, a ~1 kbp germline deletion on the opposing haplotype can also be seen (Supplementary Figure S1). Genome-wide association studies have indeed reported a susceptibility locus by *CDH18* (at 5p14.3) in hereditary colorectal cancer [50]. This highlights the capability of phasing to disentangle larger genomic events. Another example is shown in Figure 3B, where an 84 kbp deletion on chr4:q22.3 in P19 intersects several exons of the *UNC5C* gene. *UNC5C* has been suggested to be a tumor suppressor gene and reduced expression has been linked to cancer progression in CRC [51,52,53]. For P19, we also found a large inversion on chr20 (20q11.23) overlapping the *SNHG17* gene (Figure 3C). *SNHG17* codes for a long non-coding RNA whose upregulation has been related to adverse CRC prognosis [54,55].

**Figure 4:**
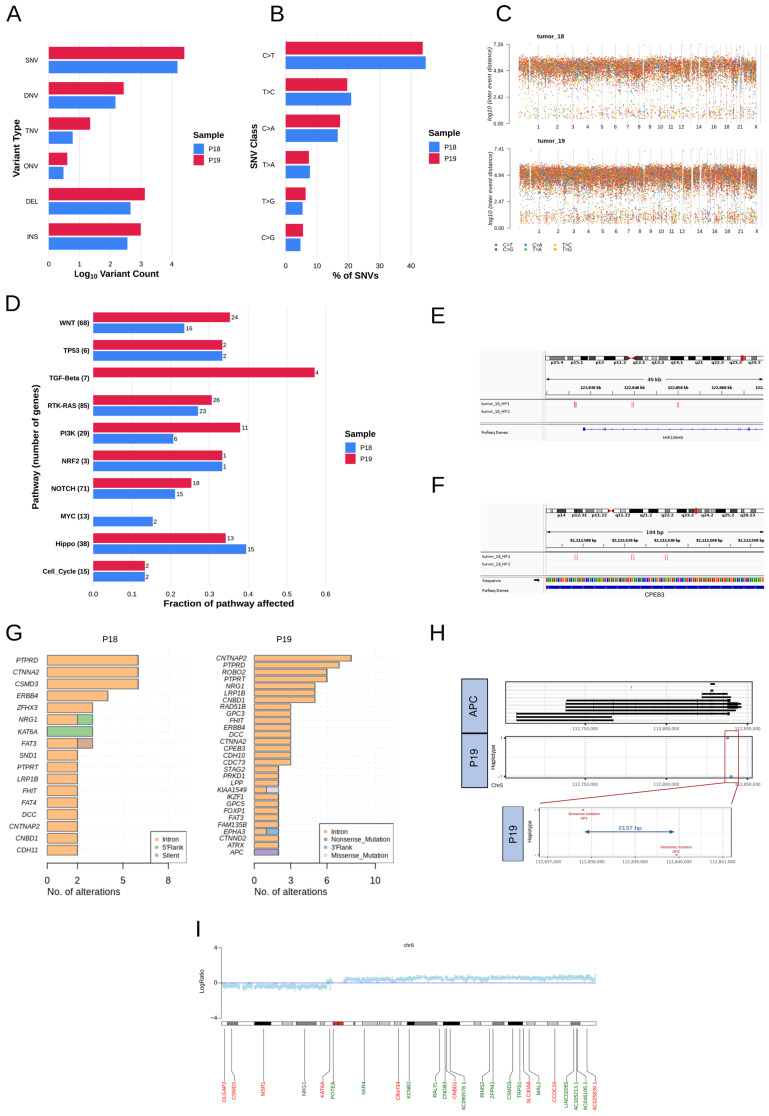
Somatic mutations analysis (**A**) Log10 number of variant types (SNV = single nucleotide variant, DNP = double nucleotide variant, TNV = triple nucleotide variant and ONV = oligo-nucleotide variant, which is a substitution in more than three consecutive nucleotides). (**B**) SNV class types (**C**) Rainfall plot for SNVs across the genome (**D**) Fraction of affected genes in the oncogenic pathways. (**E**) Highly mutated region (HMR) at (chr11: 122,026,515–122,050,025) in P18 with three mutations on the same allele. (**F**) HMR at (chr10: 92,113,497–92,113,541) in P19 with three mutations on the same allele. (**G**) Mutated genes with multiple phased SMs intersected with COSMIC. (**H**) Two nonsense mutations on different alleles of the APC gene in P19. (**I**) Chr8 in P18 with LogR copy numbers in light blue. Genes with multiple mutations on the same allele are colored red. Genes with multiple mutations on both alleles are colored green.

Small somatic mutations (SMs) were detected using joint variant calling with LANCET [44]. A total of 16,196 and 28,041 SMs passed quality filters in P18 and P19, respectively. The majority of the SMs were classified as single-nucleotide variants (SNVs) (Figure 4A), with the highest proportion of C>T (or G>A) mutations in both patients (Figure 4B). Figure 4C shows the density and the distributions of SNVs along the chromosomes, with the Y axis indicating the log10 values of inter-event distances between the SNVs. Out of all the mutations, 9967 and 17,226 SMs were associated with 6458 and 9330 unique genes as annotated by VEP [34] for P18 and P19, respectively. By performing an oncogenic pathway analysis, almost all pathways reported by the Cancer Genome Atlas (TCGA) [56] had multiple affected genes in both patients (Figure 4D) (Supplementary Table S3).

The DBS technology provides a means for resolving cis-trans co-localization of mutations. To leverage that, we first selected mutations with high haplotype scores in the tumors according to LANCET (Phredscore ≥ 30). Using this threshold, about 18% and 22% of total mutations were confidently phased to either of the alleles, in T18 and T19, respectively. We then applied sliding windows of 3 mutations throughout these SMs regions. Windows spanning up to 20 kbp between the 3 mutations were kept, and overlapping windows were merged to obtain highly mutated regions (HMRs). With this cut-off, we found 26 HMRs in P18 and 116 HMRs in P19 (Supplementary Table S4). As an example of these regions, we highlight the HMR (chr11: 122,026,515–122,050,025) in P18, where three mutations were detected on the same allele, downstream of the gene *MIR100HG* (Figure 4E), with an average distance of 7.8 kbp between the mutations. This gene is a host gene for microRNAs mir-100, let-7a-2, and mir-125b-1, which is upregulated in several cancer types and can act as an oncogenic or tumor suppressor gene [57]. Another example from P19 is an HMR on chromosome 10 (chr10: 92,113,497–92,113,541), where three mutations occurred in the same allele, and fall in an intronic region of the gene *CPEB3*. This gene has previously been reported as a tumor suppressor gene in colorectal cancer (Figure 4F) [58].

To investigate whether one or both alleles of the mutated genes are affected by the mutations, SMs labeled as intergenic regions (IGRs) were first removed from the downstream analysis, keeping 1757 and 3636 SMs in P18 and P19, respectively. Then, only SMs in genes associated with 2 or more mutations were kept. This filtration left 561 and 1600 SMs in 220 and 559 unique genes in P18 and P19, respectively, with 109 genes being common in both patients. We intersected the multi SMs genes with Cosmic CancerGeneCensus v98 [37] and found a total of 17 and 29 unique genes with more than one mutation in P18 and P19, respectively (Figure 4G) (Supplementary Table S5). Supplementary Table S5 also presents RNA expression status for these genes. A notable example here is adenomatous polyposis coli (*APC*), a tumor suppressor gene on chromosome 18 that is one of the most frequently mutated genes in colorectal cancer [59,60]. We found two somatic alterations in P19 that resulted in nonsense mutations, 2137 bp apart. Using the DBS linked-read information, we were able to show that there was one SMs on each haplotype, which suggests an elimination of normal APC function (Figure 4H).

Next, to obtain an overview of the mutated genes in correlation with the copy numbers at the chromosomal level, we integrated the two modalities into a single plot. Figure 4I shows the reported mutated genes (with 2 or more SMs) on chromosome 8 of P18. Using different color codes, the figure highlights whether the mutations reside on the same allele or both alleles. This gives a handy tool to study monoallelic and biallelic mutations in their context on the genomic localizations. In addition, Supplementary Figure S2 shows the integration of these genes’ RNA expression profiles for both patients on chromosome 8.

**Figure 5:**
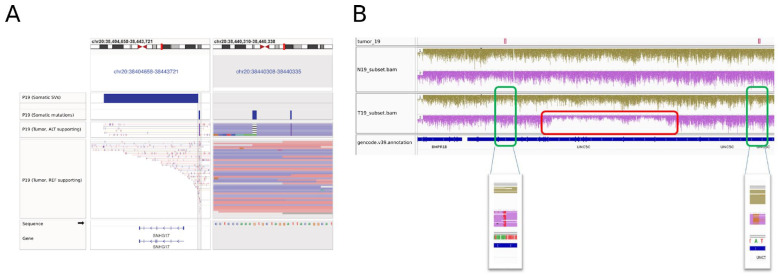
Integrated analysis of SVs and SMs in P19. (**A**) Overlap between barcodes supporting SMs proximal to inversion. The grey region in the left frame corresponds to the right frame, which shows the base calls for the corresponding barcodes. (**B**) Somatic heterozygote deletion with two SNVs on the same allele of chromosome 4q. Yellow and Purple colors indicate the two alleles. The top two alleles are normal, and the bottom two alleles are the tumor. Visualization was generated with Integrated Genome Viewer (IGV).

Lastly, we integrated the analysis from both SVs (large alterations) and SMs (small alterations) as phased data from linked-read DBS technology is a powerful method that enables this. From the previously discussed SVs, we investigated the regions surrounding the inversion (Figure 3C) and found two upstream short somatic indels on the same haplotype (Figure 5A). To validate that all these variants appear together, we extracted the barcodes supporting the somatic indels and further confirmed that they all support the inversion. In the other example, where a deletion was detected in the SV analysis (Figure 3B), we found two point mutations surrounding the deletion, all residing on the same haplotype (Figure 5B).

As a summary, in this proof-of-concept exploratory study, we demonstrate the advantage of the DBS linked-read method for resolving cancer genomes of two CRC patients. Both patients’ tumors displayed aneuploidy with many CNAs matching those common in CRC. Multiple large somatic structural variants were called and linked to germline or somatic mutations, at the same or opposing haplotypes. Several genes with multiple phased mutations were highlighted, including the CRC-related tumor suppressor gene *APC*. Together, this work presents a showcase for a comprehensive investigation of haplotype complexity in cancer genomes with the DBS linked-read method. Haplotype-resolved mutation profiles enable the detection of monoallelic and biallelic gene disruption, which is important for understanding the functional consequences of genomic alterations. This information, generated by DBS linked-read technology, may refine molecular stratification of patients and improve the precision of therapeutic decisions. In this context, application of DBS linked-read technology in larger screening cohorts may enable the identification of genes with diagnostic and prognostic relevance based not only on mutation presence but also on their allelic configuration. Building on such findings, one could envision the development of targeted DBS panels focusing on a selected set of clinically informative genes and thereby reducing the analytical complexity while retaining haplotype resolution. We have previously demonstrated the feasibility of such a targeted DBS approach for accurate haplotyping of the *HLA* gene [15], supporting its potential for scalable and cost-effective clinical applications.

## Data Availability

https://github.com/AfshinLab/CRC_article (accessed on 17 May 2026). Data available on request due to restrictions.

## Supplementary Materials

The following supporting information can be downloaded at: https://www.mdpi.com/article/10.3390/life16060874/s1, Table S1: BLR run statistics for the different DBS datasets; Table S2: Candidate SVs re-scored using NAIBR; Table S3: TCGA Pathways with the mutated genes in P18 and P19; Table S4: High mutation rate (HMR) regions in P18 and P19; Table S5: Mutated genes in P18 and P19 that have 2 or more mutations, with high allele separation score and intersect with a reference cancer database (Cosmic_CancerGeneCensus_v98_GRCh38); Figure S1: Haplotype read coverage for the loci of the deletions in P18. Yellow shade highlights the deletion locus, and red lines highlight the specific allele of the deletion; Figure S2: Integration of gene expression with genes carrying 2 or more mutations.

## References

[1] Sung H., Ferlay J., Siegel R.L., Laversanne M., Soerjomataram I., Jemal A., Bray F. (2021). Global Cancer Statistics 2020: GLOBOCAN Estimates of Incidence and Mortality Worldwide for 36 Cancers in 185 Countries. CA Cancer J. Clin..

[2] Punt C.J., Koopman M., Vermeulen L. (2017). From tumour heterogeneity to advances in precision treatment of colorectal cancer. Nat. Rev. Clin. Oncol..

[3] Chen Y., Qiu Q., She J., Yu J. (2023). Extrachromosomal circular DNA in colorectal cancer: Biogenesis, function and potential as therapeutic target. Oncogene.

[4] Nakatochi M., Kushima I., Ozaki N. (2021). Implications of germline copy-number variations in psychiatric disorders: Review of large-scale genetic studies. J. Hum. Genet..

[5] Kumar K.R., Cowley M.J., Davis R.L. (2019). Next-Generation Sequencing and Emerging Technologies. Semin. Thromb. Hemost..

[6] Hojer P., Frick T., Siga H., Pourbozorgi P., Aghelpasand H., Martin M., Ahmadian A. (2023). BLR: A flexible pipeline for haplotype analysis of multiple linked-read technologies. Nucleic Acids Res..

[7] De Maio N., Shaw L.P., Hubbard A., George S., Sanderson N.D., Swann J., Wick R., AbuOun M., Stubberfield E., Hoosdally S.J. (2019). Comparison of long-read sequencing technologies in the hybrid assembly of complex bacterial genomes. Microb. Genom..

[8] Amini S., Pushkarev D., Christiansen L., Kostem E., Royce T., Turk C., Pignatelli N., Adey A., Kitzman J.O., Vijayan K. (2014). Haplotype-resolved whole-genome sequencing by contiguity-preserving transposition and combinatorial indexing. Nat. Genet..

[9] Zhang F., Christiansen L., Thomas J., Pokholok D., Jackson R., Morrell N., Zhao Y., Wiley M., Welch E., Jaeger E. (2017). Haplotype phasing of whole human genomes using bead-based barcode partitioning in a single tube. Nat. Biotechnol..

[10] Chen Z., Pham L., Wu T.C., Mo G., Xia Y., Chang P.L., Porter D., Phan T., Che H., Tran H. (2020). Ultralow-input single-tube linked-read library method enables short-read second-generation sequencing systems to routinely generate highly accurate and economical long-range sequencing information. Genome Res..

[11] Wang O., Chin R., Cheng X., Wu M.K.Y., Mao Q., Tang J., Sun Y., Anderson E., Lam H.K., Chen D. (2019). Efficient and unique cobarcoding of second-generation sequencing reads from long DNA molecules enabling cost-effective and accurate sequencing, haplotyping, and de novo assembly. Genome Res..

[12] Meier J.I., Salazar P.A., Kucka M., Davies R.W., Dreau A., Aldas I., Box Power O., Nadeau N.J., Bridle J.R., Rolian C. (2021). Haplotype tagging reveals parallel formation of hybrid races in two butterfly species. Proc. Natl. Acad. Sci. USA.

[13] Zheng G.X., Lau B.T., Schnall-Levin M., Jarosz M., Bell J.M., Hindson C.M., Kyriazopoulou-Panagiotopoulou S., Masquelier D.A., Merrill L., Terry J.M. (2016). Haplotyping germline and cancer genomes with high-throughput linked-read sequencing. Nat. Biotechnol..

[14] Redin D., Frick T., Aghelpasand H., Kaller M., Borgstrom E., Olsen R.A., Ahmadian A. (2019). High throughput barcoding method for genome-scale phasing. Sci. Rep..

[15] Redin D., Borgstrom E., He M., Aghelpasand H., Kaller M., Ahmadian A. (2017). Droplet Barcode Sequencing for targeted linked-read haplotyping of single DNA molecules. Nucleic Acids Res..

[16] Stiller C., Aghelpasand H., Frick T., Westerlund K., Ahmadian A., Karlstrom A.E. (2019). Fast and Efficient Fc-Specific Photoaffinity Labeling To Produce Antibody-DNA Conjugates. Bioconjug. Chem..

[17] Banijamali M., Hojer P., Nagy A., Haag P., Gomero E.P., Stiller C., Kaminskyy V.O., Ekman S., Lewensohn R., Karlstrom A.E. (2022). Characterizing single extracellular vesicles by droplet barcode sequencing for protein analysis. J. Extracell. Vesicles.

[18] Testa U., Castelli G., Pelosi E. (2020). Genetic Alterations of Metastatic Colorectal Cancer. Biomedicines.

[19] Hases L., Ibrahim A., Chen X., Liu Y., Hartman J., Williams C. (2021). The Importance of Sex in the Discovery of Colorectal Cancer Prognostic Biomarkers. Int. J. Mol. Sci..

[20] Shajii A., Numanagic I., Berger B. (2018). Latent Variable Model for Aligning Barcoded Short-Reads Improves Downstream Analyses. Res. Comput. Mol. Biol..

[21] Poplin R., Chang P.C., Alexander D., Schwartz S., Colthurst T., Ku A., Newburger D., Dijamco J., Nguyen N., Afshar P.T. (2018). A universal SNP and small-indel variant caller using deep neural networks. Nat. Biotechnol..

[22] Edge P., Bafna V., Bansal V. (2017). HapCUT2: Robust and accurate haplotype assembly for diverse sequencing technologies. Genome Res..

[23] Talevich E., Shain A.H., Botton T., Bastian B.C. (2016). CNVkit: Genome-Wide Copy Number Detection and Visualization from Targeted DNA Sequencing. PLoS Comput. Biol..

[24] Olshen A.B., Bengtsson H., Neuvial P., Spellman P.T., Olshen R.A., Seshan V.E. (2011). Parent-specific copy number in paired tumor-normal studies using circular binary segmentation. Bioinformatics.

[25] Venkatraman E.S., Olshen A.B. (2007). A faster circular binary segmentation algorithm for the analysis of array CGH data. Bioinformatics.

[26] Elyanow R., Wu H.T., Raphael B.J. (2018). Identifying structural variants using linked-read sequencing data. Bioinformatics.

[27] Fang L., Kao C., Gonzalez M.V., Mafra F.A., Pellegrino da Silva R., Li M., Wenzel S.S., Wimmer K., Hakonarson H., Wang K. (2019). LinkedSV for detection of mosaic structural variants from linked-read exome and genome sequencing data. Nat. Commun..

[28] Danecek P., Bonfield J.K., Liddle J., Marshall J., Ohan V., Pollard M.O., Whitwham A., Keane T., McCarthy S.A., Davies R.M. (2021). Twelve years of SAMtools and BCFtools. Gigascience.

[29] English A.C., Menon V.K., Gibbs R.A., Metcalf G.A., Sedlazeck F.J. (2022). Truvari: Refined structural variant comparison preserves allelic diversity. Genome Biol..

[30] Jeffares D.C., Jolly C., Hoti M., Speed D., Shaw L., Rallis C., Balloux F., Dessimoz C., Bahler J., Sedlazeck F.J. (2017). Transient structural variations have strong effects on quantitative traits and reproductive isolation in fission yeast. Nat. Commun..

[31] Quinlan A.R., Hall I.M. (2010). BEDTools: A flexible suite of utilities for comparing genomic features. Bioinformatics.

[32] Belyeu J.R., Chowdhury M., Brown J., Pedersen B.S., Cormier M.J., Quinlan A.R., Layer R.M. (2021). Samplot: A platform for structural variant visual validation and automated filtering. Genome Biol..

[33] Kandoth C., Gao J., Mattioni M., Struck A., Boursin Y., Penson A., Chavan S. (2018). Mskcc/Vcf2Maf: Vcf2Maf V1, 6.16. Zenodo.

[34] McLaren W., Gil L., Hunt S.E., Riat H.S., Ritchie G.R., Thormann A., Flicek P., Cunningham F. (2016). The Ensembl Variant Effect Predictor. Genome Biol..

[35] Lu C.H., Wu C.H., Tsai M.H., Lai L.C., Chuang E.Y. (2021). MutScape: An analytical toolkit for probing the mutational landscape in cancer genomics. NAR Genom. Bioinform..

[36] Mayakonda A., Lin D.C., Assenov Y., Plass C., Koeffler H.P. (2018). Maftools: Efficient and comprehensive analysis of somatic variants in cancer. Genome Res..

[37] Sondka Z., Dhir N.B., Carvalho-Silva D., Jupe S., Madhumita, McLaren K., Starkey M., Ward S., Wilding J., Ahmed M. (2024). COSMIC: A curated database of somatic variants and clinical data for cancer. Nucleic Acids Res..

[38] Love M.I., Huber W., Anders S. (2014). Moderated estimation of fold change and dispersion for RNA-seq data with DESeq2. Genome Biol..

[39] Gel B., Serra E. (2017). karyoploteR: An R/Bioconductor package to plot customizable genomes displaying arbitrary data. Bioinformatics.

[40] Gel B.M. (2021). Miriam CopyNumberPlots: Create Copy-Number Plots Using karyoploteR Functionality. https://bioconductor.org/packages/release/bioc/html/CopyNumberPlots.html.

[41] Viswanathan S.R., Ha G., Hoff A.M., Wala J.A., Carrot-Zhang J., Whelan C.W., Haradhvala N.J., Freeman S.S., Reed S.C., Rhoades J. (2018). Structural Alterations Driving Castration-Resistant Prostate Cancer Revealed by Linked-Read Genome Sequencing. Cell.

[42] Ha G., Roth A., Khattra J., Ho J., Yap D., Prentice L.M., Melnyk N., McPherson A., Bashashati A., Laks E. (2014). TITAN: Inference of copy number architectures in clonal cell populations from tumor whole-genome sequence data. Genome Res..

[43] Narzisi G., Corvelo A., Arora K., Bergmann E.A., Shah M., Musunuri R., Emde A.K., Robine N., Vacic V., Zody M.C. (2018). Genome-wide somatic variant calling using localized colored de Bruijn graphs. Commun. Biol..

[44] Musunuri R., Arora K., Corvelo A., Shah M., Shelton J., Zody M.C., Narzisi G. (2021). Somatic variant analysis of linked-reads sequencing data with Lancet. Bioinformatics.

[45] Ried T., Knutzen R., Steinbeck R., Blegen H., Schrock E., Heselmeyer K., du Manoir S., Auer G. (1996). Comparative genomic hybridization reveals a specific pattern of chromosomal gains and losses during the genesis of colorectal tumors. Genes Chromosomes Cancer.

[46] Cancer Genome Atlas N. (2012). Comprehensive molecular characterization of human colon and rectal cancer. Nature.

[47] Maharaj S., Sharaf R., Redman R.A., Rojan A. (2022). Chromosome 20q and 13q gain in metastatic colorectal cancer: Prognostic significance and genomic correlates. J. Clin. Oncol..

[48] Van Loo P., Nordgard S.H., Lingjaerde O.C., Russnes H.G., Rye I.H., Sun W., Weigman V.J., Marynen P., Zetterberg A., Naume B. (2010). Allele-specific copy number analysis of tumors. Proc. Natl. Acad. Sci. USA.

[49] Shao X., Lv N., Liao J., Long J., Xue R., Ai N., Xu D., Fan X. (2019). Copy number variation is highly correlated with differential gene expression: A pan-cancer study. BMC Med. Genet.

[50] Dominguez-Valentin M., Therkildsen C., Da Silva S., Nilbert M. (2015). Familial colorectal cancer type X: Genetic profiles and phenotypic features. Mod. Pathol..

[51] Thiebault K., Mazelin L., Pays L., Llambi F., Joly M.O., Scoazec J.Y., Saurin J.C., Romeo G., Mehlen P. (2003). The netrin-1 receptors UNC5H are putative tumor suppressors controlling cell death commitment. Proc. Natl. Acad. Sci. USA.

[52] Bernet A., Mazelin L., Coissieux M.M., Gadot N., Ackerman S.L., Scoazec J.Y., Mehlen P. (2007). Inactivation of the UNC5C Netrin-1 receptor is associated with tumor progression in colorectal malignancies. Gastroenterology.

[53] Xing H., Wang P., Liu S., Jing S., Lin J., Yang J., Zhu Y., Yu M. (2021). A global integrated analysis of UNC5C down-regulation in cancers: Insights from mechanism and combined treatment strategy. Biomed. Pharmacother..

[54] Ma Z., Gu S., Song M., Yan C., Hui B., Ji H., Wang J., Zhang J., Wang K., Zhao Q. (2017). Long non-coding RNA SNHG17 is an unfavourable prognostic factor and promotes cell proliferation by epigenetically silencing P57 in colorectal cancer. Mol. Biosyst..

[55] Ma L., Gao J., Zhang N., Wang J., Xu T., Lei T., Zou X., Wei C., Wang Z. (2022). Long noncoding RNA SNHG17: A novel molecule in human cancers. Cancer Cell Int..

[56] Sanchez-Vega F., Mina M., Armenia J., Chatila W.K., Luna A., La K.C., Dimitriadoy S., Liu D.L., Kantheti H.S., Saghafinia S. (2018). Oncogenic Signaling Pathways in The Cancer Genome Atlas. Cell.

[57] Wu Y., Wang Z., Yu S., Liu D., Sun L. (2022). LncmiRHG-MIR100HG: A new budding star in cancer. Front. Oncol..

[58] Fang Y., Zhong Q., Wang Y., Gu C., Liu S., Li A., Yan Q. (2020). CPEB3 functions as a tumor suppressor in colorectal cancer via JAK/STAT signaling. Aging.

[59] Kandoth C., McLellan M.D., Vandin F., Ye K., Niu B., Lu C., Xie M., Zhang Q., McMichael J.F., Wyczalkowski M.A. (2013). Mutational landscape and significance across 12 major cancer types. Nature.

[60] Zhang L., Shay J.W. (2017). Multiple Roles of APC and its Therapeutic Implications in Colorectal Cancer. J. Natl. Cancer Inst..

