# Supplementary material for: Resolving the Haplotype Complexity of Colorectal Cancer Genomes with Droplet Barcode Sequencing
Source: Life (Basel). 2026 May 22;16(6):874. doi: 10.3390/life16060874 (PMC13301505; doi:10.3390/life16060874)
Supplement: Supplementary file 1 [file life-16-00874-s001.zip › life-4268901-supplementary.pdf]

Resolving the haplotype complexity of colorectal cancer genomes with droplet barcode sequencing

Supplementary information

Supplementary Table S1: BLR run statistics for the different DBS datasets

Patient	P18	P18	P19	P19
Tissue	Normal	Tumor	Normal	Tumor
Reads (M)	2188.5	1702.3	3123	3240.7
Mapped reads (%)	93.3	92.7	92.6	92.3
Median coverage	34X	28X	46X	47X
Barcodes (M)	7.9	7.0	9.0	8.5
Median molecules/barcode	4	4	4	5
Mean molecule length (kbp)	15.6	18.2	17.7	14.4
Median molecule length (kbp)	8.4	11.4	10.5	8.7
DNA in molecules >20kbp (%)	69.4	73.0	72.0	63.8
DNA in molecules >100kbp (%)	3.2	2.3	4.6	1.1
Heterozygous SNVs called (M)	2.205	2.119	2.21	2.126
Heterozygous SNVs phased (%)	99.6	99.5	99.7	99.6
N50 phase block (Mbp)	1.9	1.6	3.1	1.6
auN phase block (Mbp)	2.4	2.0	4.0	1.9
NG50 phase block (Mbp)	1.6	1.3	2.7	1.3
auNG phase block (Mbp)	2.1	1.7	3.5	1.6

Supplementary Table S2: Candidate SVs re-scored using NAIBR.

Patient	P18	P18	P19	P19
Tissue	Normal	Tumor	Normal	Tumor
SVs	1725	1716	1738	1765

Supplementary Table S3: TCGA Pathways with the mutated genes in P18 and P19.

Pathway	Gene	P18_no_mut	P19_no_mut
WNT	CHD8	1	0
WNT	LEF1	1	2
WNT	LGR4	2	0
WNT	LGR5	0	1
WNT	LRP5	0	1
WNT	LRP6	0	2
WNT	NDP	1	0
WNT	RSPO1	0	1
WNT	SFRP1	1	1
WNT	SFRP4	1	1
WNT	TCF7L1	1	1
WNT	TLE4	0	3
WNT	WIF1	1	0
WNT	ZNRF3	0	1
WNT	FZD3	0	2
WNT	FZD6	0	1
WNT	FZD7	0	1
WNT	FZD9	1	0
WNT	WNT1	1	0
WNT	WNT10A	1	0
WNT	WNT3A	1	1
WNT	WNT4	0	1
WNT	WNT5A	0	1
WNT	WNT7A	0	1
WNT	WNT8B	0	1
WNT	WNT9B	0	1
WNT	APC	1	3
WNT	DKK2	1	1
WNT	GSK3B	1	2
WNT	RNF43	0	1
WNT	TCF7L2	1	2
WNT	CHD4	1	0
TP53	MDM2	1	1
TP53	ATM	1	0
TP53	RPS6KA3	0	1
TGF-Beta	TGFBR1	0	1
TGF-Beta	TGFBR2	0	1
TGF-Beta	ACVR1B	0	1

TGF-Beta	SMAD2	0	4
RTK-RAS	ABL1	2	2
RTK-RAS	EGFR	0	1
RTK-RAS	ERBB4	7	8
RTK-RAS	PDGFRB	0	1
RTK-RAS	MET	0	2
RTK-RAS	FGFR1	0	1
RTK-RAS	FLT3	0	3
RTK-RAS	ALK	4	8
RTK-RAS	ROS1	1	1
RTK-RAS	IGF1R	1	3
RTK-RAS	NTRK2	1	1
RTK-RAS	NTRK3	5	1
RTK-RAS	SOS1	1	0
RTK-RAS	KRAS	1	1
RTK-RAS	BRAF	1	2
RTK-RAS	RAF1	0	1
RTK-RAS	NF1	1	2
RTK-RAS	RASA1	0	1
RTK-RAS	CBL	3	2
RTK-RAS	CBLB	3	2
RTK-RAS	INSR	0	1
RTK-RAS	INSRR	0	1
RTK-RAS	SOS2	0	1
RTK-RAS	SHC3	1	0
RTK-RAS	SHC4	0	1
RTK-RAS	RASGRP2	1	0
RTK-RAS	RAPGEF1	2	0
RTK-RAS	RAPGEF2	1	0
RTK-RAS	RASGRF1	2	0
RTK-RAS	RASGRF2	1	0
RTK-RAS	ICMT	0	1
RTK-RAS	MRAS	0	1
RTK-RAS	ARHGAP35	0	1
RTK-RAS	RASA2	1	0
RTK-RAS	RASA3	0	3
RTK-RAS	RASAL2	2	0
RTK-RAS	DAB2IP	1	0
RTK-RAS	SHOC2	1	0
RTK-RAS	KSR2	5	0
RTK-RAS	JAK2	0	1
PI3K	AKT3	2	0
PI3K	DEPTOR	2	0

PI3K	INPP4B	7	5
PI3K	MAPKAP1	1	1
PI3K	MTOR	0	2
PI3K	NPRL3	0	1
PI3K	PIK3CA	0	1
PI3K	PIK3R1	1	1
PI3K	PIK3R3	0	1
PI3K	PTEN	0	1
PI3K	RICTOR	1	1
PI3K	RPTOR	0	1
PI3K	RPS6	0	1
NRF2	CUL3	1	2
NOTCH	CNTN6	1	2
NOTCH	HEYL	1	0
NOTCH	KDM5A	0	1
NOTCH	NOTCH2	0	2
NOTCH	NOTCH4	0	1
NOTCH	ITCH	0	3
NOTCH	FHL1	1	0
NOTCH	THBS2	0	2
NOTCH	CUL1	0	1
NOTCH	NCOR1	0	1
NOTCH	NCOR2	0	1
NOTCH	NUMB	0	1
NOTCH	MAML3	2	3
NOTCH	MFNG	1	0
NOTCH	CIR1	1	0
NOTCH	CNTN1	2	3
NOTCH	MAML1	2	0
NOTCH	MAML2	4	3
NOTCH	PSEN1	2	0
NOTCH	RBPJ	2	2
NOTCH	RBPJL	0	1
NOTCH	SNW1	1	0
NOTCH	CTBP2	0	2
NOTCH	ADAM17	1	0
NOTCH	DLK1	0	1
NOTCH	DNER	2	2
NOTCH	DTX4	1	0
MYC	MXI1	2	0
MYC	MYC	1	0
MYC	MYCN	1	0

Hippo	STK4	0	2
Hippo	STK3	2	5
Hippo	LATS1	0	1
Hippo	MOB1A	0	1
Hippo	MOB1B	1	0
Hippo	YAP1	0	1
Hippo	TEAD1	1	0
Hippo	TEAD4	1	0
Hippo	PTPN14	1	2
Hippo	NF2	1	0
Hippo	TAOK1	1	0
Hippo	TAOK3	1	0
Hippo	CRB1	1	2
Hippo	HMCN1	1	8
Hippo	HIPK2	0	2
Hippo	FAT1	1	2
Hippo	FAT3	4	6
Hippo	FAT4	4	0
Hippo	DCHS2	1	3
Hippo	WTIP	1	1
Cell_Cycle	CDKN1B	1	0
Cell_Cycle	RB1	1	4
Cell_Cycle	E2F3	0	2

Supplementary Table S4: High mutation rate (HMR) regions in P18 and P19.

P18

	Chromosome	Start	End	Width (bp)	Number of mutations	Average in-ter-mutation distance
1	chr1	189473377	189524305	50929	3	16976.33
2	chr2	41337620	41397183	59564	3	19854.67
3	chr2	153642251	153699862	57612	3	19204.00
4	chr3	78253132	78280528	27397	3	9132.33
5	chr3	132980325	133001604	21280	3	7093.33
6	chr3	145245439	145299318	53880	3	17960.00
7	chr4	133644408	133693761	49354	3	16451.33
8	chr7	49464370	49509536	45167	3	15055.67
9	chr7	55682587	55690823	8237	3	2745.67
10	chr8	42053128	42054091	964	3	321.33
11	chr8	57268920	57323345	54426	3	18142.00
12	chr8	86644699	86663108	18410	5	3682.00
13	chr8	113754551	113784443	29893	4	7473.25
14	chr8	137552903	137607073	54171	3	18057.00
15	chr9	23920615	23959086	38472	3	12824.00
16	chr9	40920790	40920799	10	3	3.33
17	chr9	102603800	102627618	23819	3	7939.67
18	chr11	55504205	55563521	59317	3	19772.33
19	chr11	122026515	122050025	23511	3	7837.00
20	chr12	98062623	98112215	49593	3	16531.00
21	chr12	129479128	129533644	54517	3	18172.33
22	chr13	56292087	56349890	57804	3	19268.00
23	chr13	62450374	62498625	48252	3	16084.00
24	chrX	26195867	26272403	76537	4	19134.25
25	chrX	52325235	52363989	38755	3	12918.33
26	chrX	94885032	94885042	11	3	3.67

P19

	Chromosome	Start	End	Width (bp)	Number of mutations	Average in-ter-mutation distance
1	chr1	72368584	72389248	20665	3	6888.33
2	chr1	80513552	80568491	54940	3	18313.33
3	chr1	103882185	103932125	49941	3	16647.00
4	chr1	105243181	105301176	57996	3	19332.00
5	chr1	189763279	189780095	16817	3	5605.67
6	chr1	190304529	190348732	44204	3	14734.67
7	chr1	227848192	227866377	18186	3	6062.00
8	chr1	236962849	236985014	22166	3	7388.67

9	chr2	12985946	13001022	15077	3	5025.67
10	chr2	57080630	57114430	33801	3	11267.00
11	chr2	57463389	57511632	48244	3	16081.33
12	chr2	76269258	76310625	41368	3	13789.33
13	chr2	81404202	81440732	36531	3	12177.00
14	chr2	143519304	143569250	49947	3	16649.00
15	chr2	144861816	144873653	11838	3	3946.00
16	chr2	153556883	153571513	14631	3	4877.00
17	chr2	208749181	208791413	42233	3	14077.67
18	chr3	28949429	28982260	32832	3	10944.00
19	chr3	82687570	82767647	80078	4	20019.50
20	chr3	107270570	107287182	16613	3	5537.67
21	chr3	180343261	180377096	33836	3	11278.67
22	chr3	195723266	195723282	17	3	5.67
23	chr4	58798056	58853293	55238	3	18412.67
24	chr4	96650587	96700638	50052	3	16684.00
25	chr4	117245680	117279091	33412	3	11137.33
26	chr4	131096658	131151602	54945	4	13736.25
27	chr4	171745235	171801322	56088	4	14022.00
28	chr4	177057609	177104795	47187	4	11796.75
29	chr5	3192501	3232458	39958	3	13319.33
30	chr5	5798104	5827416	29313	3	9771.00
31	chr5	24597472	24656058	58587	3	19529.00
32	chr5	27118111	27177616	59506	3	19835.33
33	chr5	27859152	27916609	57458	4	14364.50
34	chr5	85812022	85860748	48727	3	16242.33
35	chr5	91716682	91737803	21122	3	7040.67
36	chr5	121256447	121296628	40182	4	10045.50
37	chr5	154944829	155000927	56099	3	18699.67
38	chr6	10686061	10718586	32526	3	10842.00
39	chr6	124062932	124074379	11448	3	3816.00
40	chr7	9884011	9907211	23201	3	7733.67
41	chr7	10793211	10833576	40366	3	13455.33
42	chr7	41980812	42026037	45226	3	15075.33
43	chr7	42085831	42155858	70028	5	14005.60
44	chr7	52815838	52905533	89696	4	22424.00
45	chr7	68761767	68798806	37040	4	9260.00
46	chr7	69116018	69157796	41779	3	13926.33
47	chr7	113334681	113374510	39830	3	13276.67
48	chr7	136295915	136346398	50484	3	16828.00
49	chr7	142736493	142761448	24956	3	8318.67
50	chr8	3853329	3884095	30767	3	10255.67
51	chr8	4859496	4887914	28419	3	9473.00
52	chr8	83847092	83898687	51596	4	12899.00
53	chr8	88599772	88644848	45077	4	11269.25
54	chr8	114993612	115042968	49357	3	16452.33
55	chr8	137018822	137056256	37435	3	12478.33
56	chr9	12135945	12153975	18031	3	6010.33

57	chr9	17242357	17264477	22121	3	7373.67
58	chr9	103763447	103798947	35501	3	11833.67
59	chr10	7576901	7652936	76036	4	19009.00
60	chr10	82563091	82622218	59128	4	14782.00
61	chr10	92113497	92113541	45	3	15.00
62	chr10	97236120	97282103	45984	3	15328.00
63	chr10	106112115	106135441	23327	9	2591.89
64	chr10	130673091	130731588	58498	3	19499.33
65	chr11	25181167	25214653	33487	3	11162.33
66	chr11	40248499	40304967	56469	4	14117.25
67	chr11	40628395	40685825	57431	3	19143.67
68	chr11	98186721	98207185	20465	4	5116.25
69	chr12	34569163	34601673	32511	3	10837.00
70	chr12	37293710	37343457	49748	3	16582.67
71	chr12	85993841	86052963	59123	3	19707.67
72	chr12	126529003	126568805	39803	3	13267.67
73	chr13	19238464	19285230	46767	3	15589.00
74	chr13	19387221	19425548	38328	3	12776.00
75	chr13	36433643	36483748	50106	3	16702.00
76	chr13	58538084	58624468	86385	6	14397.50
77	chr13	65463874	65512344	48471	3	16157.00
78	chr13	66683057	66737396	54340	4	13585.00
79	chr13	68378871	68409806	30936	3	10312.00
80	chr13	78198961	78238204	39244	3	13081.33
81	chr13	93868826	93884333	15508	3	5169.33
82	chr14	37604567	37644972	40406	3	13468.67
83	chr14	47666698	47694299	27602	3	9200.67
84	chr14	47852770	47902333	49564	3	16521.33
85	chr14	82634746	82662749	28004	3	9334.67
86	chr14	83300913	83345687	44775	3	14925.00
87	chr14	87238280	87293517	55238	3	18412.67
88	chr16	8340703	8365300	24598	3	8199.33
89	chr16	13445549	13467444	21896	3	7298.67
90	chr16	61643378	61686462	43085	4	10771.25
91	chr16	61902307	61958979	56673	3	18891.00
92	chr16	64910673	64932584	21912	3	7304.00
93	chr16	87122561	87179729	57169	3	19056.33
94	chr17	50682588	50682908	321	4	80.25
95	chr18	15381343	15403064	21722	3	7240.67
96	chr20	29705327	29734330	29004	3	9668.00
97	chr20	48761994	48777853	15860	3	5286.67
98	chr21	13017519	13025980	8462	3	2820.67
99	chr21	21767301	21781657	14357	3	4785.67
100	chr21	22496153	22570053	73901	5	14780.20
101	chrX	5267327	5304969	37643	3	12547.67
102	chrX	30769270	30786083	16814	3	5604.67
103	chrX	34111287	34169395	58109	3	19369.67
104	chrX	66460789	66499266	38478	4	9619.50

105	chrX	67803252	67839197	35946	3	11982.00
106	chrX	76073796	76116860	43065	3	14355.00
107	chrX	87560326	87590702	30377	3	10125.67
108	chrX	88799302	88856451	57150	3	19050.00
109	chrX	91562508	91614431	51924	3	17308.00
110	chrX	91998059	92037228	39170	3	13056.67
111	chrX	92485290	92535256	49967	3	16655.67
112	chrX	126122346	126156505	34160	3	11386.67
113	chrX	141724931	141772318	47388	3	15796.00
114	chrX	144264282	144341311	77030	4	19257.50
115	chrX	145072405	145106867	34463	3	11487.67
116	chrX	145212230	145237737	25508	3	8502.67

Supplementary Table S5: Mutated genes in P18 and P19 that have 2 or more mutations, with high allele separation score and intersect with a reference cancer database (Cosmic_CancerGeneCensus_v98_GRCh38)

P18

Gene	Chromosome	Start Position	End Position	HP	Variant Classification	Log2 Ratio (T-exp/N-exp)
CTNNA2	chr2	79369179	79369179	1	Intron	-0.549
CTNNA2	chr2	79445540	79445540	1	Intron	
CTNNA2	chr2	79450796	79450796	1	Intron	
CTNNA2	chr2	80253668	80253668	2	Intron	
CTNNA2	chr2	80568541	80568541	1	Intron	
CTNNA2	chr2	80626468	80626468	1	Intron	
LRP1B	chr2	141067504	141067504	1	Intron	0.341
LRP1B	chr2	142107659	142107659	1	Intron	
ERBB4	chr2	211501505	211501505	2	Intron	-0.534
ERBB4	chr2	211693438	211693438	1	Intron	
ERBB4	chr2	211783462	211783462	1	Intron	
ERBB4	chr2	211887896	211887896	2	Intron	
FHIT	chr3	60043366	60043366	1	Intron	-0.886
FHIT	chr3	60448139	60448139	2	Intron	
FAT4	chr4	125336098	125336098	1	Intron	0.905
FAT4	chr4	125354729	125354729	2	Intron	
SND1	chr7	127981976	127981976	1	Intron	-0.313
SND1	chr7	128002521	128002521	2	Intron	
CNTNAP2	chr7	146571800	146571800	1	Intron	-2.015
CNTNAP2	chr7	147205710	147205710	2	Intron	
NRG1	chr8	31637996	31637996	1	5'Flank	-2.706
NRG1	chr8	32005567	32005567	2	Intron	
NRG1	chr8	32698469	32698469	2	Intron	

KAT6A	chr8	42053128	42053128	2	5'Flank	-0.100
KAT6A	chr8	42053256	42053256	2	5'Flank	
KAT6A	chr8	42054090	42054090	2	5'Flank	
CNBD1	chr8	87326230	87326230	2	Intron	NA
CNBD1	chr8	87345026	87345026	2	Intron	
CSMD3	chr8	112496159	112496159	2	Intron	0.744
CSMD3	chr8	112544699	112544699	1	Intron	
CSMD3	chr8	112699154	112699154	1	Intron	
CSMD3	chr8	112980761	112980761	2	Intron	
CSMD3	chr8	113117261	113117261	2	Intron	
CSMD3	chr8	113213080	113213080	2	Intron	
PTPRD	chr9	8533861	8533861	1	Intron	0.349
PTPRD	chr9	9512445	9512445	1	Intron	
PTPRD	chr9	9592305	9592305	1	Intron	
PTPRD	chr9	9915455	9915455	1	Intron	
PTPRD	chr9	9956829	9956829	2	Intron	
PTPRD	chr9	10093202	10093202	1	Intron	
FAT3	chr11	92364486	92364486	1	Intron	2.064
FAT3	chr11	92651864	92651864	1	Intron	
FAT3	chr11	92810060	92810060	2	Silent	
CDH11	chr16	64952854	64952854	2	Intron	1.415
CDH11	chr16	65117332	65117332	1	Intron	
ZFHX3	chr16	73540405	73540405	2	Intron	0.293
ZFHX3	chr16	73780591	73780591	2	Intron	
ZFHX3	chr16	73890421	73890421	2	Intron	
DCC	chr18	52363069	52363069	1	Intron	0.449
DCC	chr18	52363081	52363081	1	Intron	
PTPRT	chr20	42212012	42212012	1	Intron	0.550
PTPRT	chr20	42720223	42720223	1	Intron	

P19

Gene	Chromosome	Start Position	End Position	HP	Variant Classification	Log2 Ratio (T-exp/N-exp)
CDC73	chr1	193138885	193138885	1	Intron	-0.453
CDC73	chr1	193220259	193220259	2	Intron	
CDC73	chr1	193230852	193230852	2	Intron	
CTNNA2	chr2	80290395	80290395	1	Intron	-1.206
CTNNA2	chr2	80552105	80552105	2	Intron	
CTNNA2	chr2	80590041	80590041	2	Intron	
LRP1B	chr2	141265914	141265914	1	Intron	-9.939
LRP1B	chr2	141577939	141577939	2	Intron	
LRP1B	chr2	141597376	141597376	1	Intron	
LRP1B	chr2	141700135	141700135	1	Intron	
LRP1B	chr2	142019596	142019596	1	Intron	
ERBB4	chr2	211463891	211463891	2	Intron	-12.805
ERBB4	chr2	211944521	211944521	2	Intron	
ERBB4	chr2	212047861	212047861	2	Intron	
FHIT	chr3	60616578	60616578	1	Intron	-1.103
FHIT	chr3	61165728	61165728	1	Intron	
FHIT	chr3	61188498	61188498	2	Intron	
FOXP1	chr3	71184768	71184768	2	Intron	-0.731
FOXP1	chr3	71461756	71461756	1	Intron	
ROBO2	chr3	76071516	76071516	1	Intron	-0.291
ROBO2	chr3	76203782	76203782	2	Intron	
ROBO2	chr3	76315138	76315138	2	Intron	
ROBO2	chr3	76495094	76495094	1	Intron	
ROBO2	chr3	77016308	77016308	1	Intron	
ROBO2	chr3	77087032	77087032	2	Intron	
EPHA3	chr3	89339313	89339313	1	Intron	0.545
EPHA3	chr3	89485386	89485386	2	3'Flank	

LPP	chr3	188425294	188425294	1	Intron	-0.826
LPP	chr3	188794990	188794990	2	Intron	
CTNND2	chr5	11390607	11390607	2	Intron	-10.864
CTNND2	chr5	11582340	11582340	1	Intron	
CDH10	chr5	24513317	24513317	1	Intron	-6.630
CDH10	chr5	24597472	24597472	2	Intron	
CDH10	chr5	24603170	24603170	2	Intron	
APC	chr5	112837805	112837805	1	Nonsense_Mutation	-0.410
APC	chr5	112839942	112839942	2	Nonsense_Mutation	
IKZF1	chr7	50325528	50325528	1	Intron	-3.107
IKZF1	chr7	50363309	50363309	1	Intron	
KIAA1549	chr7	138916861	138916861	2	Missense_Mutation	2.166
KIAA1549	chr7	138944417	138944417	1	Intron	
CNTNAP2	chr7	146551129	146551129	1	Intron	1.333
CNTNAP2	chr7	146715287	146715287	1	Intron	
CNTNAP2	chr7	147041857	147041857	2	Intron	
CNTNAP2	chr7	147138845	147138845	1	Intron	
CNTNAP2	chr7	147140322	147140322	1	Intron	
CNTNAP2	chr7	147381407	147381407	1	Intron	
CNTNAP2	chr7	147969135	147969135	2	Intron	
CNTNAP2	chr7	148262868	148262868	1	Intron	
NRG1	chr8	31795045	31795045	1	Intron	-2.403
NRG1	chr8	31920694	31920694	2	Intron	
NRG1	chr8	32282018	32282018	2	Intron	
NRG1	chr8	32316523	32316523	1	Intron	
NRG1	chr8	32510319	32510319	2	Intron	
CNBD1	chr8	87241430	87241430	2	Intron	NA
CNBD1	chr8	87275777	87275777	2	Intron	
CNBD1	chr8	87422546	87422546	1	Intron	
CNBD1	chr8	87481263	87481263	1	Intron	

CNBD1	chr8	87528443	87528443	1	Intron	
FAM135B	chr8	138345319	138345319	2	Intron	-5.178
FAM135B	chr8	138415853	138415853	1	Intron	
PTPRD	chr9	9074088	9074088	1	Intron	-0.684
PTPRD	chr9	9874410	9874410	2	Intron	
PTPRD	chr9	10067986	10067986	1	Intron	
PTPRD	chr9	10289800	10289800	1	Intron	
PTPRD	chr9	10296199	10296199	1	Intron	
PTPRD	chr9	10405256	10405256	1	Intron	
PTPRD	chr9	10430313	10430313	1	Intron	
CPEB3	chr10	92113497	92113497	2	Intron	-1.558
CPEB3	chr10	92113524	92113524	2	Intron	
CPEB3	chr10	92113540	92113540	2	Intron	
FAT3	chr11	92466594	92466594	2	Intron	0.161
FAT3	chr11	92688821	92688821	1	Intron	
GPC5	chr13	91736968	91736968	2	Intron	-6.630
GPC5	chr13	92701097	92701097	2	Intron	
PRKD1	chr14	29744811	29744811	2	Intron	0.438
PRKD1	chr14	30017816	30017816	1	Intron	
RAD51B	chr14	68260022	68260022	2	Intron	0.876
RAD51B	chr14	68323710	68323710	2	Intron	
RAD51B	chr14	68521521	68521521	1	Intron	
DCC	chr18	52393941	52393941	1	Intron	0.707
DCC	chr18	52405756	52405756	2	Intron	
DCC	chr18	53042648	53042648	2	Intron	
PTPRT	chr20	42081447	42081447	1	Intron	-5.178
PTPRT	chr20	42280187	42280187	1	Intron	
PTPRT	chr20	42430643	42430643	2	Intron	
PTPRT	chr20	42783933	42783933	1	Intron	

PTPRT	chr20	42969076	42969076	2	Intron	
PTPRT	chr20	43076195	43076195	2	Intron	
ATRX	chrX	77546093	77546093	1	Intron	-0.514
ATRX	chrX	77630225	77630225	1	Intron	
STAG2	chrX	124001151	124001151	2	Intron	-0.026
STAG2	chrX	124080247	124080247	2	Intron	
GPC3	chrX	133684757	133684757	1	Intron	-2.900
GPC3	chrX	133912840	133912840	2	Intron	
GPC3	chrX	133925409	133925409	2	Intron	

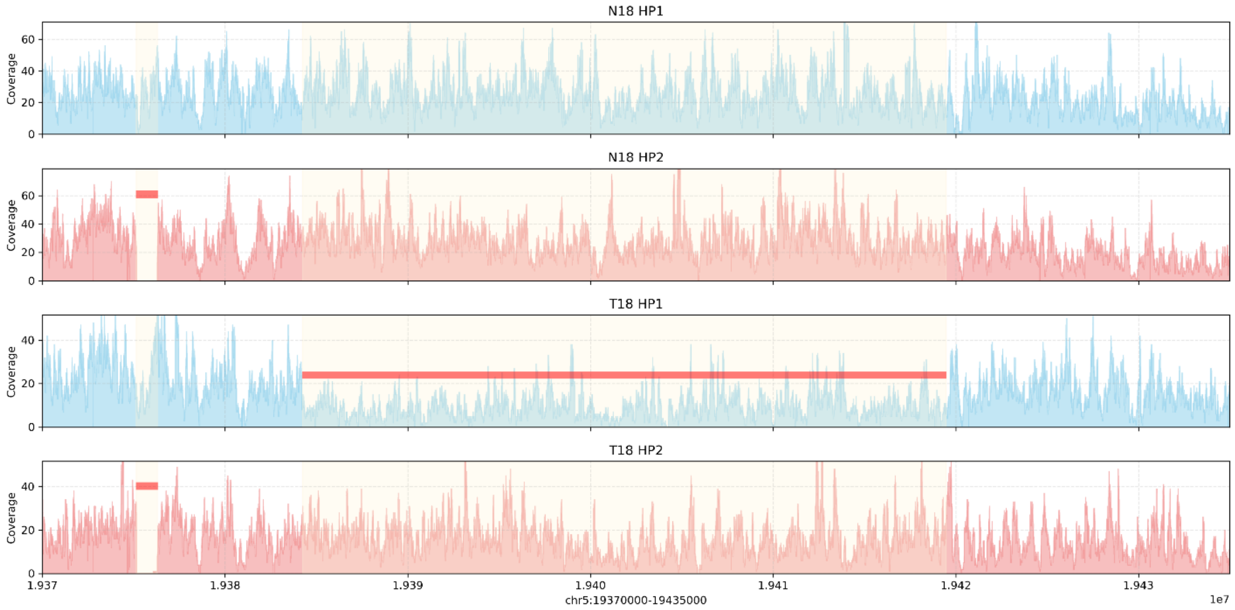

Supplementary Figure S1: Haplotype read coverage for the loci of the deletions in P18.Yellow shade highlights the deletion locus, and red lines highlight the specific allele of the deletion. (Note: HP2 was assigned to the allele with the germline deletion).

P18

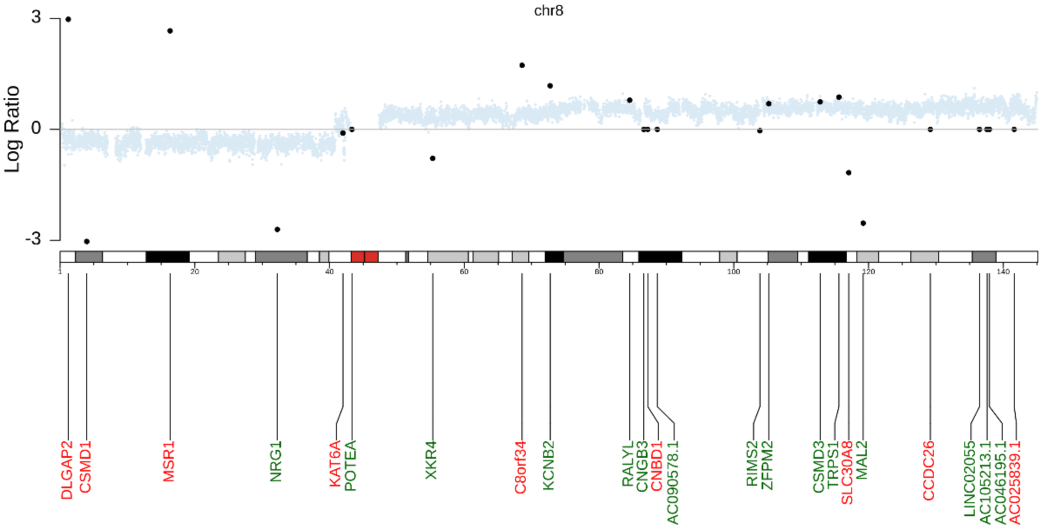

P19

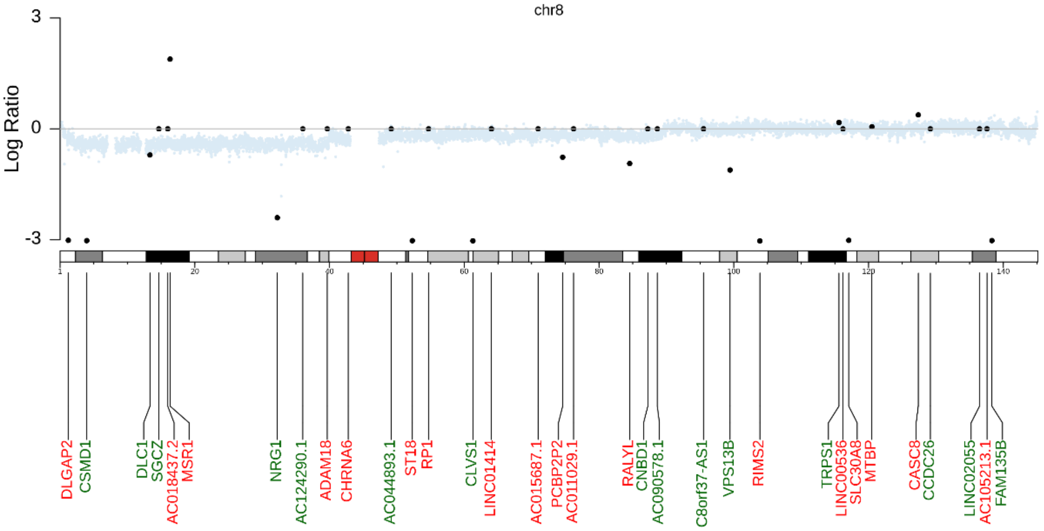

Supplementary Figure S2: Integration of gene expression with genes carrying 2 or more mutations. Black dots represent log2 ratio (tumor/normal) of the corresponding normalized RNA expression. The values are set to max 3 and min -3. The values were set to 0 if the genes were filtered out in the RNA analysis. Gene name is colored in red if its mutations reside on the same allele, and in green when the mutations reside on both alleles. The light blue represents the log ratio of copy number values, as reported by Titan.
